# Circular RNA_0006014 promotes breast cancer progression through sponging miR-885-3p to regulate NTRK2 and PIK3/AKT pathway

**DOI:** 10.18632/aging.203996

**Published:** 2022-04-05

**Authors:** Xiqian Zhou, Wei Jian, Qifeng Luo, Wenfang Zheng, Xiaochong Deng, Xuehui Wang, Oyungerel Borkhuu, Changle Ji, Dengfeng Li, Lin Fang

**Affiliations:** 1 Department of Breast and Thyroid Surgery, Shanghai Tenth People's Hospital, Tongji University School of Medicine, Shanghai 200072, China

**Keywords:** breast cancer, hsa_circ_0006014, NTRK2, progression, miR-885-3p

## Abstract

Breast cancer is the most common cancer in women worldwide. Numerous reports have demonstrated that circRNAs play an essential role in regulating the biological characteristics of breast cancer. However, there are currently no reports regarding the role of hsa_circ_0006014 in breast cancer. In this study, qRT-PCR was used to detect the expression of hsa_circ_0006014 and related genes. MTT, colony formation and Transwell assays were used to explore the potential biological functions of hsa_circ_0006014 in breast cancer cells. Western blotting was used to explore the potential molecular mechanisms involving hsa_circ_0006014. *In vivo* experiments were used to evaluate the influence of hsa_circ_0006014 on animal tumors. In this study, we found higher expression of hsa_circ_0006014 in breast tumor samples than in matched adjacent normal samples, and its expression was positively correlated with histological grade (grade iii). Phenotypically, hsa_circ_0006014 promoted the proliferation of MDA-MB-231 and MCF-7 breast cancer cells. Mechanistically, there were confirmed binding sites between hsa_circ_0006014 and miR-885-3p, and hsa_circ_0006014 promoted breast cancer cell proliferation partially by sponging miR-885-3p and influenced CDK2/CCNE1 and CDK4/6/CCND1. Furthermore, we found that hsa_circ_0006014 regulated NTRK2 through miR-885-3p to modulate the PIK3/AKT signaling pathway. Our results demonstrated that hsa_circ_0006014 promotes breast cancer progression by sponging miR-885-3p to regulate the NTRK2/PIK3CA/AKT axis.

## INTRODUCTION

Breast cancer (BC) is due to uncontrolled proliferation of mammary epithelial cells under the action of various carcinogenic factors. There were 2.26 million new cases worldwide in 2020, which ranks as the highest incidence rate of 11.7% according to the International Agency for Research on Cancer (IARC) [1, 2]. In addition, 680,000 women died of breast cancer in 2020, which is the highest number of female cancer-related deaths in the world, accounting for 15.5% of all cancer deaths [2]. Similarly, breast cancer had the highest number of new cases of cancer among Chinese women in 2020 with a rate of 19.9% [1] and results in a major cancer burden in China. To date, high endogenous estrogen levels [3], family history of breast cancer [4, 5], endometriosis [6], genetic mutations [7, 8], etc. have been proven to be risk factors for breast cancer. However, the exact cause of breast cancer remains unknown. Therefore, identification of the probable mechanism of tumorigenesis and therapeutic targets for breast cancer is urgently needed.

Circular RNAs (circRNAs) are stable noncoding small RNAs due to their covalently bonded loop structure without 5′ and 3′ polyadenylation and a poly-A tail and were initially thought to be the product of splicing errors of endogenous RNAs [9]. It has been reported that circRNAs participate in various biological processes because of their unique structures, and the most researched function of circRNAs is sponging microRNAs (miRNAs) and inhibiting miRNA activity and therefore regulating the expression of target genes [10]. For example, circ0061825 was reported to regulate breast cancer progression and EMT by sponging miR-326 and targeting TFF1 [11]; circ0002178 sponged miR-328-3p to regulate cellular viability, metabolism and angiogenesis in breast cancer [12].

MiRNAs are small endogenous RNAs with lengths of approximately 19–25 nucleotides that negatively regulate gene expression by directly binding to target genes and are involved in various biological functions, including cancer cell proliferation, metastasis, and progression [13]. One study reported that exosomal miR-7641 could drive breast cancer progression and metastasis via the transportation of exosomes [14]; miR-142-3p could suppress breast cancer malignancy by targeting HMGA2 [15] I In addition, many studies have reported that miRNAs are key regulators of different cancer types and are promising targets for cancer treatment and diagnostic tools [16]. For instance, exosomal miRNA-205 could promote chemoresistance and tumorigenesis through E2F1 in breast cancer, and targeting exosomal miRNA-205 may be a treatment strategy to reduce cancer chemoresistance [17]; One study reported that five plasma miRNAs (let-7b-5p, miR-122-5p, miR-146b-5p, miR-210-3p and miR-215-5p) could serve as promising biomarkers for breast cancer detection [18].

Neurotrophic receptor tyrosine kinase 2 (NTRK2), also known as Trk-β, encodes a member of the NTRK family of proteins [19]. NTRK fusions were reported to occur in a variety of solid tumors, especially in secretory carcinomas, such as breast cancer [20]. NTRK family proteins bind with neurotrophins, which are transmembrane bridges that cause dimerization and subsequent phosphorylation of the neurotrophin protein, thus activating several key downstream intracellular pathways, such as the MAPK, PIK3, PLC-gamma and PI3/AKT pathways [20]. The fusion type of NTRK2 is specifically decreased in breast cancer [21] while NTRK2 itself is closely related to breast cancer; it is related to sleep disorders after surgery and increases the incidence of side effects after chemotherapy [22]. The PI3K/AKT signaling pathway is one of the most important cellular signaling pathways and plays a critical role in cell survival, cell cycle progression and cancer angiogenesis [23]. Various studies have reported that miRNAs can target the major components of the PI3K/AKT signaling pathway and act either as oncogenes by activating the PI3K/AKT pathway or as tumor suppressors by suppressing this pathway [24].

In our study, we found that the expression of hsa_circ_0006014 (circ_6014) was higher in breast tumor tissues than in paracarcinoma tissues, and functional assays revealed that circ_6014 could affect the proliferation, migration and invasion of breast cancer. Moreover, we observed that overexpression of circ_6014 led to the downregulation of miR-885-3p expression and upregulation of NTRK2 expression *in vitro*, suggesting that circ_6014 acts as a ceRNA. Furthermore, inhibition of circ_6014 inhibited the PIK3/AKT pathway. Our study indicates that circ_6014 could sponge miR-885-3p and thus regulate the NTRK2/PIK3/AKT pathway in breast cancer, which we hope could be a new therapeutic strategy for breast cancer in the future.

## RESULTS

### Characteristics of circ_6014 in breast cancer patients and cells

**Figure 1:**
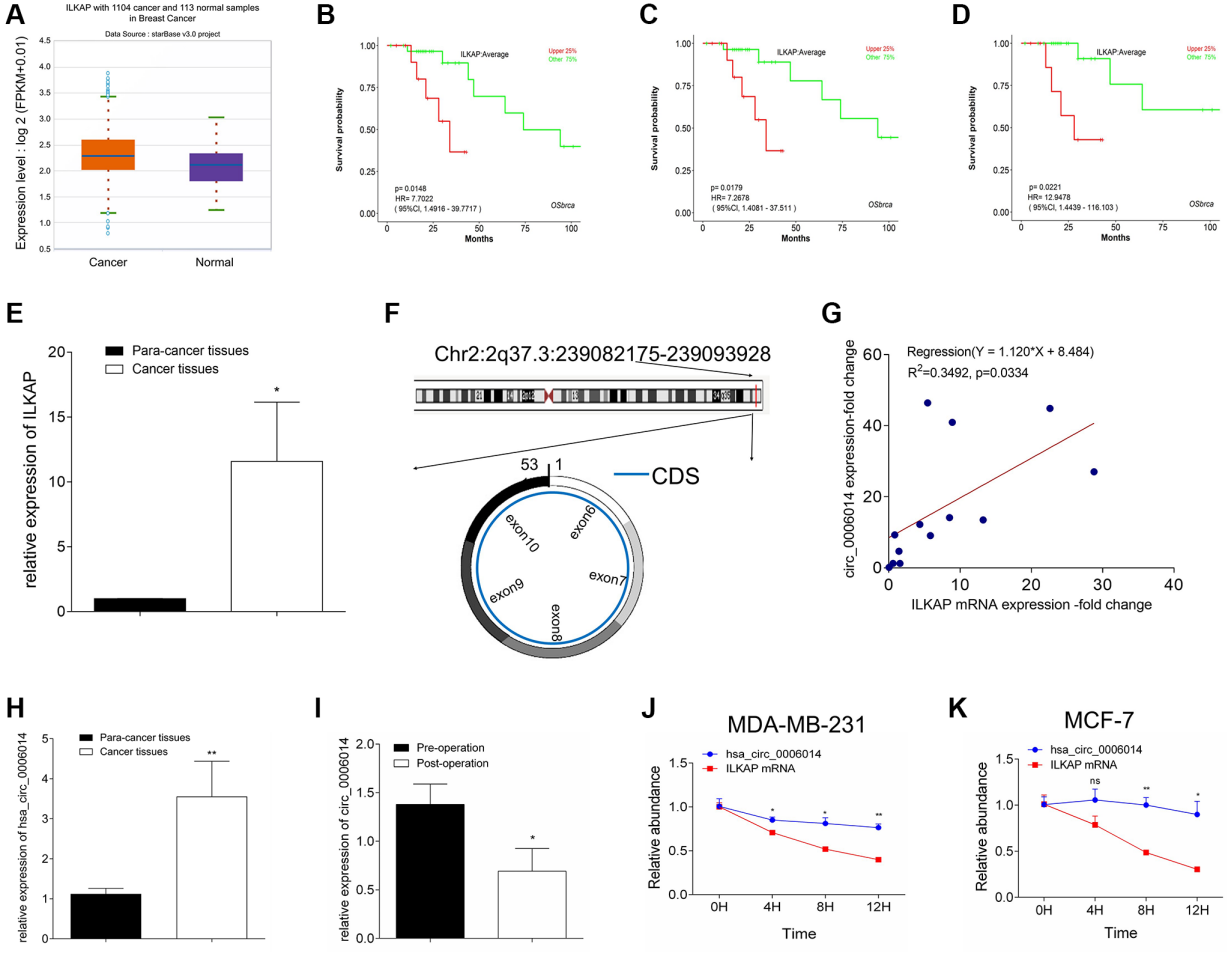
**Analysis of circ_6014 in breast cancer.** (**A**) Relative expression of the host gene circ_6014 in breast cancer according to ENCORI. (**B**–**D**) Kaplan-Meier survival curve showed higher ILKAP expression indicated worse patient prognosis in GSE10885 (**B**), GSE10893 (**C**), and GSE2741 databases (**D**). (**E**) Relative expression of ILKAP in breast cancer and adjacent noncancer tissues. (**F**) The simplified graphic of the spliced location of circ_6014 from its host gene and the 6-10 exons that constituted this circRNA. (**G**) The positive linear correlation between the mRNA level of ILKAP and the expression level of circ_6014 in breast cancer tissues. (**H**) circ_6014 was more highly expressed in breast cancer tissues compared to adjacent non-cancer tissues. (**I**) Relative expression of circ_6014 between the patients’ preoperative and postoperative plasma and downregulation of its expression after the operation. (**J**, **K**) Relative abundance of circ_6014 and its host gene ILKAP under treatment with actinomycin D for different times. The abundance of ILKAP decreased, while that of circ_6014 remained invariant. ^*^p < 0.05, ^**^p < 0.01, ^***^p < 0.001.

According to the ENCORI database (http://starbase.sysu.edu.cn/index.php) (Figure 1A), we found that ILKAP (Gene ID: 80895, location: 2q37.3, chr2:239082174-239093928, https://www.ncbi.nlm.nih.gov/gene/80895) is highly expressed in breast cancer samples compared with adjacent normal samples. In addition, the Kaplan-Meier survival curve from LOGpc (http://bioinfo.henu.edu.cn/DatabaseList.jsp) revealed that patients with higher ILKAP expression levels had a worse overall survival rate than those with lower ILKAP expression levels from GSE10885 (*p* = 0.0148) (Figure 1B), GSE10893 (*p* = 0.0179) (Figure 1C), and GSE2741 (*p* = 0.0221) (Figure 1D). qRT-PCR of the relative expression of ILKAP between breast cancer tissues and paired adjacent normal tissues from Shanghai Tenth Hospital corresponded to the results of the database (Figure 1E) (Student’s *t* test, *p* < 0.05). Our research focused on noncoding RNA regulation in breast cancer. Therefore, the circRNA circ_6014, which is from the host gene ILKAP and cyclized in the head-to-tail direction from exon 6 to exon 10, attracted our attention (http://circrna.org/cgi-bin/simplesearch.cgi) (Figure 1F).

Importantly, the fold changes of circ_6014 in breast cancer tissues were positively correlated with those of ILKAP (Y = 1.120X + 8.484, R^2^ = 0.3492, *p* = 0.334; Figure 1G). Similarly, we found that circ_6014 has higher expression in breast cancer tissues, which corresponded to the results that ILKAP is highly expressed in breast cancer tissues (Figure 1H) (Student’s *t* test, *p* < 0.05). Furthermore, we determine the relative expression of circulating circ_6014 in blood between preoperative and postoperative plasma from BC patients, and the relative expression level of circ_6014 apparently decreased after the tumor was removed (Figure 1I) (Student’s *t* test, *p* < 0.05). Our data showed that higher expression of circ_6014 was related to a worse histological grade (grade iii) (Table 1) (Fisher’s exact test, *p* < 0.05). To confirm the existence and stability of circ_6014 in breast cancer, we treated MDA-MB-231 and MCF-7 cells with actinomycin D, which can inhibit transcription, and observed that the level of linear ILKAP was decreased, while the circ_6014 level was maintained over time (Figure 1J, 1K). Our results confirmed that circ_6014 was present in breast cancer and highly expressed in breast cancer tissues and was positively correlated with clinical-pathological parameters and worse overall survival.

**Table 1:** Statistical analysis of correlations between clinical pathological parameters characteristics and expression of hsa_circ_0006014 in BC tissues (Fisher exact test).

**Clinical pathological parameters**		**circ_6014 expression**		***P***
		**high**	**low**	
Age	<50	2	0	1.000
	≥50	9	5	
Tumor size (mm)	<20	4	1	1.000
	≥20	7	4	
Lymphonodus metastasis	Yes	4	2	1.000
	No	7	3	
TNM status	I~II	9	3	0.547
	III~IV	2	2	
Histological grading	i~ii	2	4	0.036
	iii	9	1	
ER status	+	5	2	1.000
	−	6	3	
PR status	+	5	1	0.588
	−	6	4	
HER-2 status	+	1	0	1.000
	−	10	5	
Ki67 status	<30%	3	2	1.000
	≥30%	8	3	
Molecular classification	TNBC	5	3	1.000
	the others	6	2	

### circ_6014 promoted breast cancer cellular proliferation

**Figure 2:**
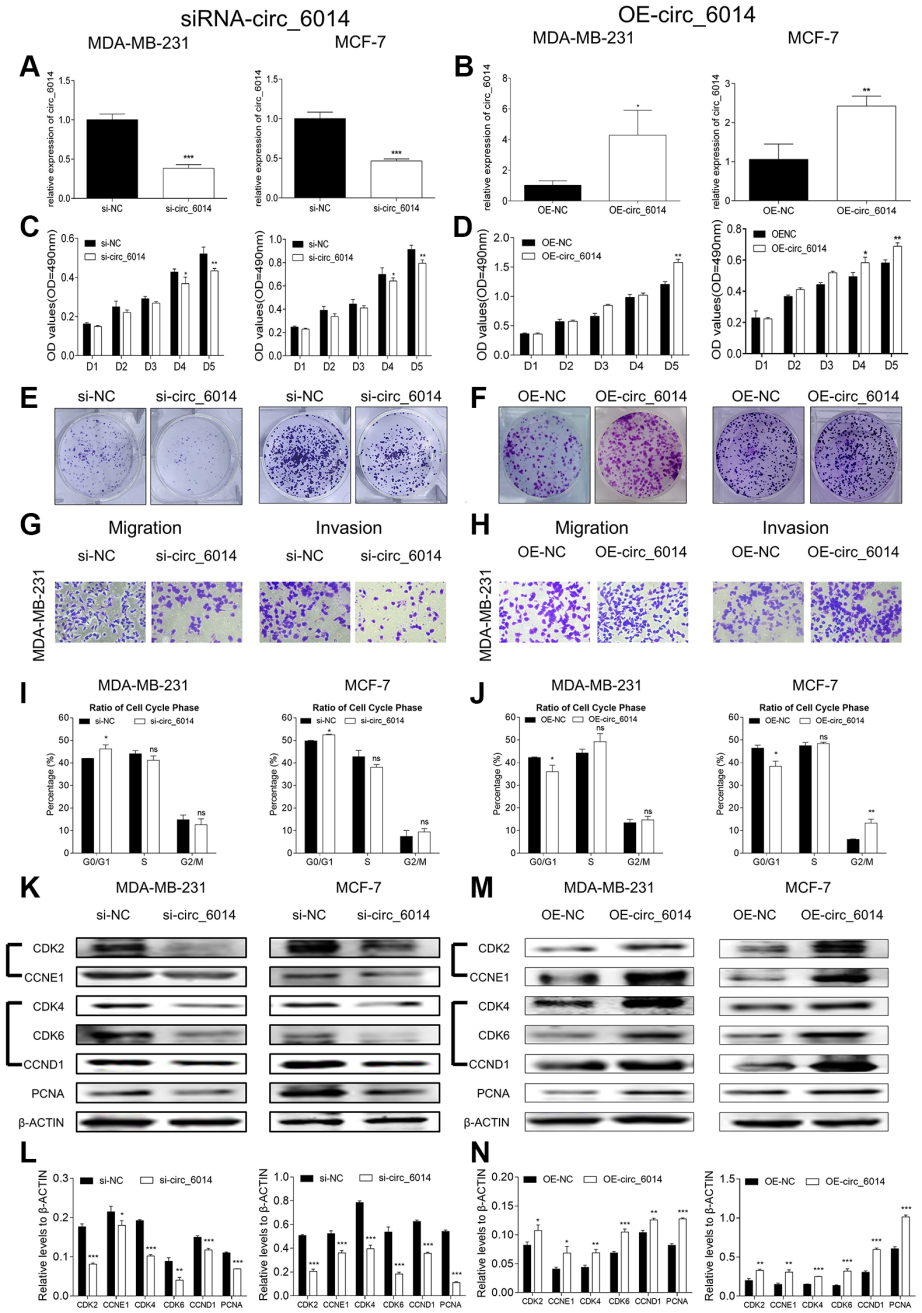
**Biological functions of circ_6014 in breast cancer cells.** (**A**, **B**) Expression levels of circ_6014 in both MDA-MB-231 and MCF-7 cells after transfection with the second small interfering RNA and circ_6014 plasmids. (**C**, **D**) MTT assays of both cell lines showed that the proliferation was inhibited (**C**) or promoted (**D**) with decreased or overexpressed levels of circ_6014. (**E**, **F**) Colony formation assays showed that the down- or upregulated level of circ_6014 suppressed (**E**) or enhanced (**F**) the cellular colony-forming capacity. (**G**, **H**) Transwell assays showed that migration and invasion were suppressed (**G**) by downregulating circ_6014 expression and promoted (**H**) by upregulating circ_6014 expression. (**I**, **J**) The percentage of cell cycle distribution of the G0/G1, S, and G2/M phases with down- or upregulation of circ_6014 expression in both breast cancer cells; circ_6014 could affect the progression of G0/G1 phase. (**K**) Protein levels of CDK2, CCNE1, CDK4, CDK6, CCND1, and PCNA in MDA-MB-231 and MCF-7 cells decreased after decreasing the expression of circ_6014. (**L**) Columns were used to quantify the cell cycle protein expression levels in (**K**) relative to β-actin. (**M**) Protein levels of CDK2, CCNE1, CDK4, CDK6, CCND1, and PCNA in MDA-MB-231 and MCF-7 cells increased after increasing the expression of circ_6014. (**N**) Columns were used to quantify the cell cycle protein expression levels in (**M**) relative to β-actin. ^*^p < 0.05, ^**^p < 0.01, ^***^p < 0.001.

MDA-MB-231 and MCF-7 cells were used, and three circ_6014 siRNAs were separately transfected into both cell lines to eliminate off-target effects (Supplementary Figure 1A, 1B). Circ_6014 plasmids were also transfected. Then, qRT-PCR was used to verify the efficiency of siRNA and the constructed plasmid (Figure 2A, 2B) (Student’s *t* test, *p* < 0.05). The MTT results showed that downregulation of circ_6014 expression suppressed the proliferation of MDA-MB-231 and MCF7 cells and that upregulation of circ_6014 expression promoted breast cancer cell proliferation in a time-dependent manner (Figure 2C, 2D). Additionally, colony formation and Transwell assays confirmed the biological function of circ_6014 in proliferation, migration and invasion (Figure 2E–2H and Supplementary Figure 1C, 1D) (Student’s *t* test, *p* < 0.05).

Then, we used flow cytometry to detect the cell cycle progression of both breast cancer cell lines. The results indicated that downregulation of circ_6014 expression arrested the cell cycle in the G0/G1 phase (Figure 2I and Supplementary Figure 1E, 1F) (Student’s *t* test, *p* < 0.05), and upregulation of circ_6014 expression promoted this phase (Figure 2J and Supplementary Figure 1G, 1H) (Student’s *t* test, *p* < 0.05). Then, we detected the potential protein levels of cell cycle and proliferation markers after transfection of circ_6014 siRNAs and plasmids. We found that decreased expression of circ_6014 decreased the protein expression levels of PCNA, CDK2/CCNE1, and CDK4/6/CCND1, and overexpression of circ_6014 increased the expression of these proteins in breast cancer cells (Figure 2K–2N) (Student’s *t* test, *p* < 0.05). Thus, circ_6014 could influence breast cancer cellular proliferation via regulation of the cell cycle through changes in CDK2/CCNE1 and CDK4/6/CCND1 cell cycle proteins and regulation of the G0/G1 phase of the cell cycle.

### Filtrating miRNAs that bind to circ_6014 in breast cancer

**Figure 3:**
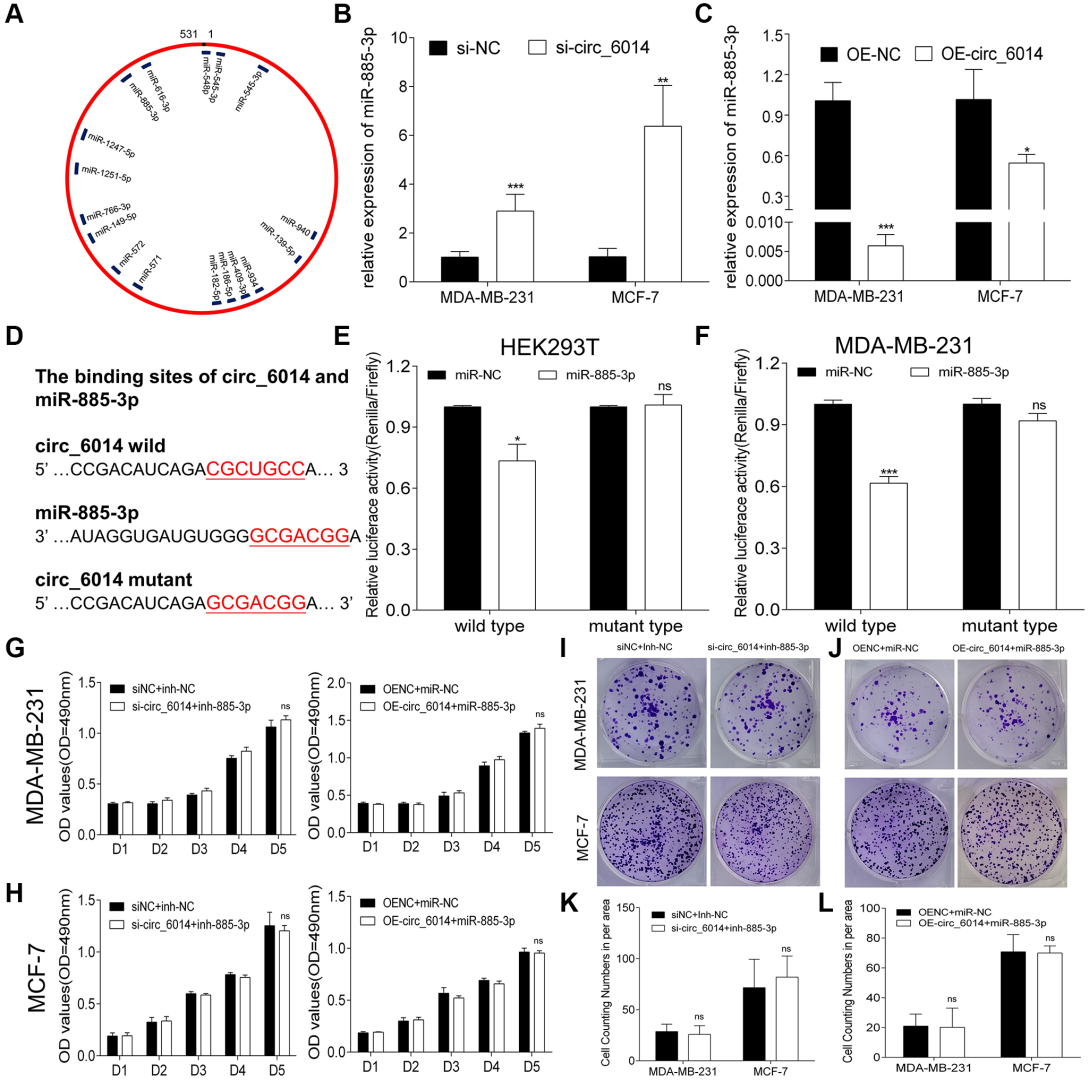
**Circ_6014 regulated the expression of miR-885-3p**. (**A**) MiRNAs that have binding sites with circ_6014 according to two databases (miRanda and RNA hybrid). (**B**, **C**) The expression level of miR-885-3p increased (**B**) or decreased (**C**) with the down- or upregulation of circ_6014 expression. (**D**) The predicted wild-type binding sites and designed mutant-type binding sites of miR-885-3p and circ_6014. (**E**, **F**) Dual-luciferase reporter assays conducted in HEK293T and MDA-MB-231 cells with miR-885-3p and circ_6014 suggested that miR-885-3p decreased the relative activity of luciferase reporters of the wild-type group. (**G**, **H**) Transfection of si-NC + miRNA-inhibitor-NC, si- circ_6014 + inhibitor-miR-885-3p or OE-NC + miRNA-mimics-NC, or OE- circ_6014 + miR-885-3p and the absorbance of formazan solution at 490 nm showed that the decrease or increase in miR-885-3p could restrain the impact caused by down- or upregulating circ_6014 expression. (**I**, **J**) Colony formation of both cell lines in six-well plates transfected with si-NC + miRNA-inhibitor-NC, si-circ_6014 + inhibitor-miR-885-3p or OE-NC + miRNA-mimics-NC, or OE-circ_6014 + miR-885-3p. (**K**, **L**) Columns used to compare the difference between cell counting numbers per area revealed that miR-885-3p could reverse the changes in biological function caused by circ_6014. ^*^p < 0.05, ^**^p < 0.01, ^***^p < 0.001.

To identify the miRNAs that may have a potential binding site of circ_6014, we examined four circRNA databases (circular RNA interactome, ENCORI, miRanda, and RNA hybrid). The schematic binding sites of 17 miRNAs and circ_6014, which were randomly selected from two of four databases, are shown in Figure 3A. A total of eight miRNAs that were predicted in three of four databases (Supplementary Table 1) were selected and analyzed in the literature of https://pubmed.gov/ to identify potential functions in cancer. According to the logical relationships of ceRNAs, four miRNAs were chosen for further experiments (Supplementary Table 2). qRT-PCR was performed to verify whether these four miRNAs are sponged by circ_6014 in breast cancer cells (Supplementary Figure 2A, 2B). We found that after changing the expression of circ_6014, miR-885-3p was changed accordingly compared to that of a negative control group in both cell lines (Figure 3B, 3C) (Student’s *t* test, *p* < 0.05).

### circ_6014 interacted with miR-885-3p

According to the above results, miR-885-3p had the highest possibility of directly binding to circ_6014. Hence, luciferase reporter assays were used to prove this hypothesis. Therefore, two special vectors involving the wild-type and mutant-type binding domains of the circ_6014 predicted binding region were constructed (Figure 3D). Compared with the negative control, miR-885-3p obviously decreased the relative activity (Renilla/firefly) of luciferase reporters of the wild-type group, while the mutant-type group revealed no change (Student’s *t* test, *p* < 0.05) in accordance with the predictions (Figure 3E). Then, we cotransferred the vectors and mimics into MDA-MB-231 cells and obtained results similar to those in HEK293T cells (Figure 3F). These results verified that miR-885-3p can directly bind to circ_6014.

To confirm whether the promotional function of circ_6014 in BC occurs by binding miR-885-3p, we performed rescue experiments. The siRNA of circ_6014 described above was cotransfected with the inhibitor of miR-885-3p, while the circ_6014 plasmid was cotransfected with the mimics of miR-885-3p. Their negative controls were cotransfected at the same time into both MDA-MB-231 and MCF-7 cells. Functional assays revealed that a decrease or increase in miR-885-3p could restrain the impact caused by the interference or increase in circ_6014 (Figure 3G–3L). Taken together, our results demonstrated that circ_6014 can interact with miR-885-3p in breast cancer cells.

### miR-885-3p inhibited breast cancer cell proliferation and motility ability

**Figure 4:**
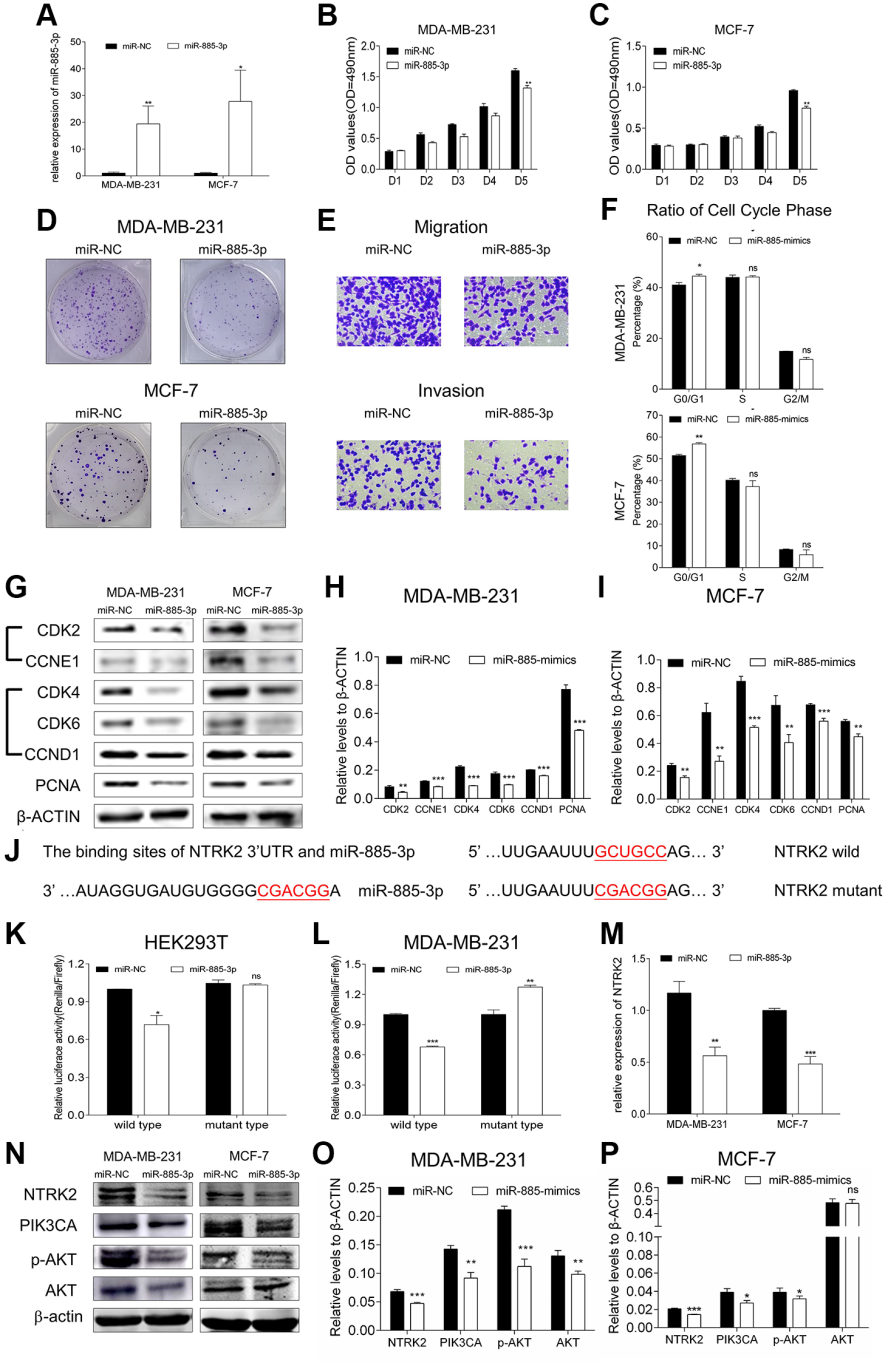
**MiR-885-3p suppressed the proliferation of breast cancer and targeted NTRK2 to affect the PIK3CA/AKT signaling pathway.** (**A**) The transfection efficiency of miR-885-3p mimics in MDA-MB-231 and MCF-7 cells. (**B**, **C**) MTT assays of both cell lines showed that the proliferation was inhibited by overexpression of miR-885-3p. (**D**) Colony formation assays showed that overexpression of miR-885-3p suppressed both cellular colony-forming capacities. (**E**) The migration and invasion of MDA-MB-231 cells were suppressed after overexpressing miR-885-3p. (**F**) Histogram of the percentage of cell cycle distribution of G0/G1, S, and G2/M phases with overexpression of miR-885-3p in both breast cancer cells; miR-885-3p could arrest both cells in G0/G1 phase. (**G**) Protein levels of CDK2, CCNE1, CDK4, CDK6, CCND1, and PCNA in MDA-MB-231 and MCF-7 cells decreased after increasing the expression of miR-885-3p. (**H**, **I**) Columns were used to quantify the cell cycle protein expression levels in (**I**) relative to β-actin. (**J**) The predicted wild-type binding sites and designed mutant-type binding sites of miR-885-3p and NTRK2. (**K**, **L**) Dual-luciferase reporter assays conducted in HEK293T and MDA-MB-231 cells with miR-885-3p and NTRK2 suggested that miR-885-3p decreased the relative activity of luciferase reporters of the wild-type group. (**M**) Relative expression of NTRK2 decreased in the MDA-MB-231 and MCF-7 cells overexpressing miR-885-3p. (**N**) The protein levels of NTRK2, PIK3CA, and p-AKT decreased in the two cell lines treated with miR-885-3p mimics, while those of AKT remained unchanged. (**O**, **P**) Columns were used to quantify the protein expression levels relative to β-actin. ^*^p < 0.05, ^**^p < 0.01, ^***^p < 0.001.

Since circ_6014 could interact with miR-885-3p in breast cancer, we explored its potential functions in breast cancer cells. We transfected mimics of miR-885-3p and its negative control into both BC cell lines and used qRT-PCR to ensure the overexpression efficiency (Figure 4A) (Student’s *t* test, *p* < 0.05). Then, functional experiments were performed to validate the function of miR-885-3p. MTT proliferation assays of the two cell lines suggested that miR-885-3p inhibited the proliferation of BC cells *in vitro* (Figure 4B, 4C). Furthermore, the colony formation and Transwell assays indicated that BC cellular proliferation and motility were depressed (Figure 4D, 4E and Supplementary Figure 2C) (Student’s *t* test, *p* < 0.05). Then, we used flow cytometry to detect the cell cycle progression of both breast cancer cell lines after increasing the expression level of miR-885-3p, and the results suggested that the cell cycle was arrested at the G0/G1 phase (Figure 4F and Supplementary Figure 1I, 1J) (Student’s *t* test, *p* < 0.05). In addition, western blotting indicated that miR-885-3p suppressed the progression of breast cancer cells by downregulating the expression level of PCNA and the expression levels of CDK2/CCNE1 and CDK4/6/CCND1 (Figure 4G–4I) (Student’s *t* test, *p* < 0.05). Our results suggested that miR-885-3p could suppress the proliferation of breast cancer cells, partially by suppressing the expression levels of CDK2/CCNE1 and CDK4/6/CCND1 and arresting the cell cycle in the G0/G1 phase.

### miR-885-3p targeted NTRK2/PIK3CA/AKT pathway

In accordance with the four miRNAs that may bind to circ_6014, we used reliable databases (TargetScan/TS, miRDB) to identify the mRNAs that may have binding sites with miRNAs by using the keywords miR-885-3p and miR-766-3p and drew a Venn diagram to determine whether the potential mRNAs identified from the two databases overlapped (Supplementary Figure 2D). Then, we selected nine mRNA candidates from the four overlapping areas that were proven to promote the progression of cancer from published articles (Supplementary Table 3). We then tested these candidates by downregulating the expression of circ_6014 in MDA-MB-231 cells and found that NTRK2 was the most likely candidate (Supplementary Figure 2E).

Then, we chose a binding site that has highest context score percentile and constructed luciferase reporter plasmids according to the binding sites of miR-885-3p and the 3′-UTR of NTRK2 mRNA (Figure 4J and Supplementary Figure 2F). The two vectors were cotransfected into both HEK293T cells and MDA-MB-231 cells with mimics of miR-885-3p or its negative control. The results indicated that miR-885-3p could target NTRK2 in both cell lines (Figure 4K, 4L) (Student’s *t* test, *p* < 0.05). In addition, overexpression of miR-885-3p suppressed the mRNA and protein levels of NTRK2 in MDA-MB-231 and MCF-7 cells. The qRT-PCR results for the relative expression of NTRK2 in the miR-885-3p overexpression group also indicated that the miR-885-3p target NTRK2 had strongly downregulated expression (Figure 4M) (Student’s *t* test, *p* < 0.05), which proved the close relationship between miR-885-3p and NTRK2. The protein-protein interaction database STRING (https://string-db.org/cgi/input.pl) was applied to explore whether the protein translated from NTRK2-mRNA can interact with other proteins. Therefore, we detected the expression and phosphorylation of AKT after overexpression of miR-885-3p. We found that the expression of PIK3CA and phosphorylation of AKT (threonine 308) were obviously weakened, while that of AKT remained unchanged in both BC cell lines. (Figure 4N–4P). Taken together, our results indicated that miR-885-3p could target NTRK2 and suppress the expression of PIK3CA and phosphorylation of AKT (threonine 308).

### circ_6014 regulated PIK3CA/AKT pathway through NTRK2

**Figure 5:**
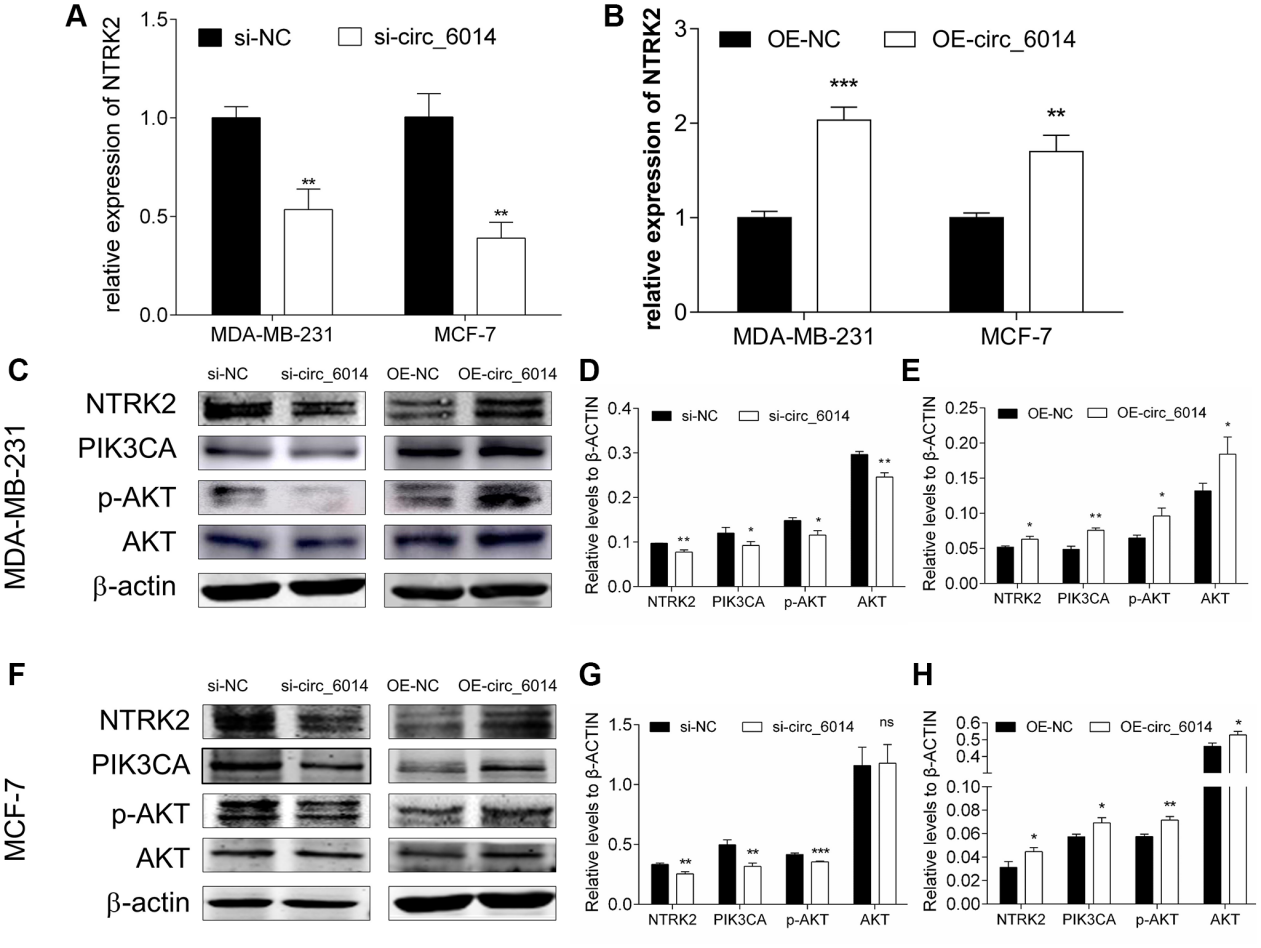
**The influence of circ_6014 on its target gene NTRK2 and the downstream PIK3CA/AKT signaling pathway.** (**A**, **B**) Relative expression of NTRK2 in the MDA-MB-231 and MCF-7 cells transfected with siRNAs or circ_6014 plasmids. (**C**) Protein levels of NTRK2, PIK3CA, p-AKT, and AKT in MDA-MB-231 cells after decreasing or increasing the expression of circ_6014. (**D**, **E**) Quantitative statistics of protein expression levels in MDA-MB-231 cells. (**F**) Protein levels of NTRK2, PIK3CA, p-AKT, and AKT in MCF-7 cells after decreasing or increasing the expression of circ_6014. (**G**, **H**) Quantitative statistics of protein expression levels in MCF-7 cells. ^*^p < 0.05, ^**^p < 0.01, ^***^p < 0.001.

As the relative expression level of the assumed target gene NTRK2 was verified in the circ_6014-silenced MDA-MB-231 cells, we further determined that in another BC cell line, MCF-7, the results indicated that NTRK2 was apparently decreased (Figure 5A) (Student’s *t* test, *p* < 0.05). Overexpression of circ_6014 increased the expression of NTRK2 in both BC cell lines (Figure 5B) (Student’s *t* test, *p* < 0.05). The positive correlation between the expression level of NTRK2 and that of circ_6014 was further confirmed by western blots of both MDA-MB-231 and MCF-7 cells. Likewise, the protein expression of PIK3CA and phosphorylation of AKT (threonine 308) were obviously weakened by decreasing the expression of circ_6014 and strengthened by increasing the expression of circ_6014 in both BC cell lines (Figure 5C–5H). Our results indicated that NTRK2 is one of the target genes of circ_6014 and governs the biological function of circ_6014 by affecting the PIK3CA/AKT pathway.

### Knockdown of circ_6014 suppressed breast cancer tumor growth *in vivo*

**Figure 6:**
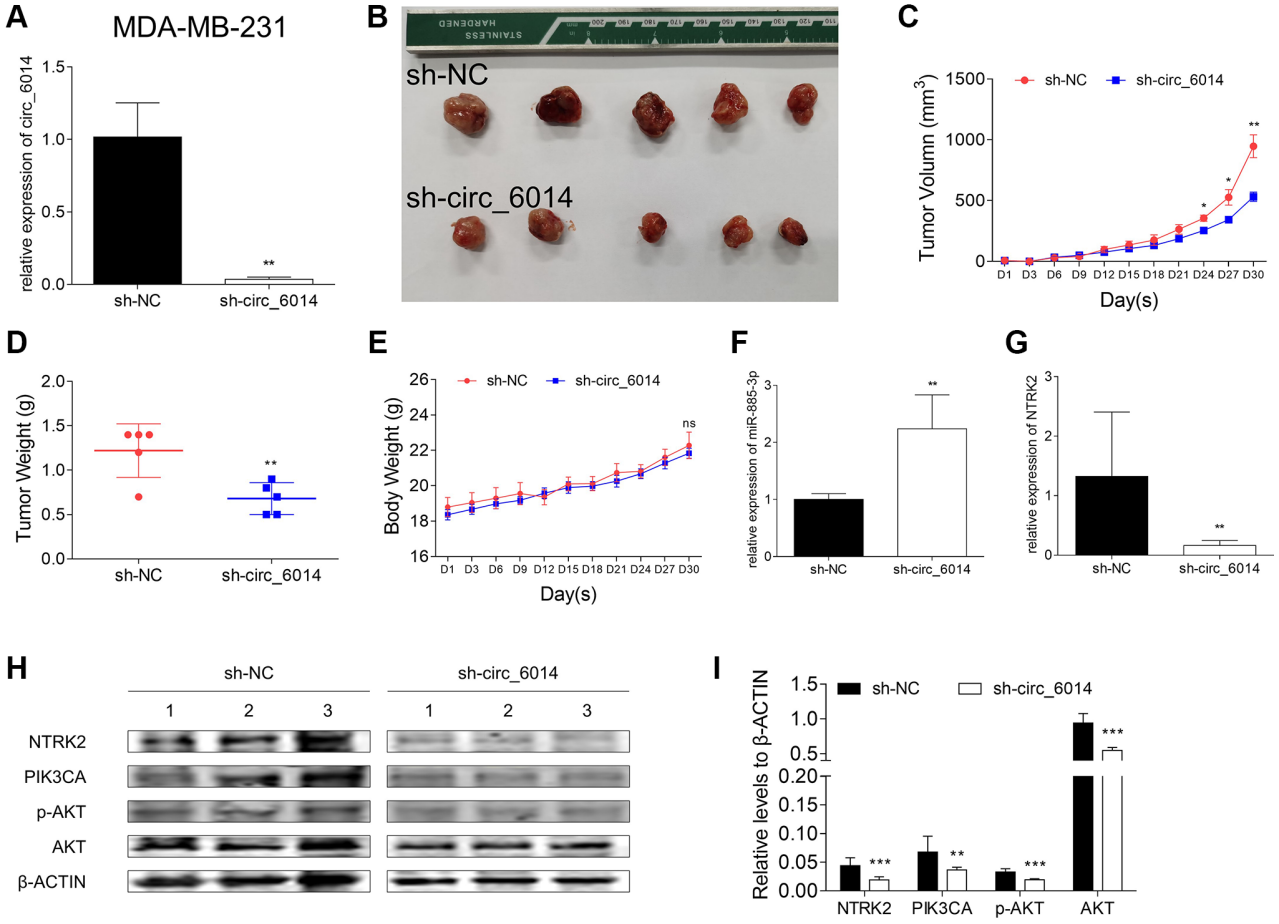
**Xenograft experiments in female nude mice with circ_6014 knockdown.** (**A**) Expression level of circ_6014 in stable circ_6014 knockdown MDA-MB-231 cells. (**B**) Photo of individual tumors. (**C**) The size of tumors of each group varied with time, and that of the experimental group was smaller. (**D**) The comparison of the tumor weight between the circ_6014 knockdown group and the negative control group and that of the experimental group was lighter. (**E**) The weight of the mice in the two groups varied with time. (**F**) The expression level of miR-885-3p increased in individual tumors of the stable circ_6014 knockdown mice compared with those of the negative control mice. (**G**) The expression level of NTRK2 decreased in individual tumors of the stable circ_6014 knockdown mice compared with the negative control mice. (**H**) Protein levels of NTRK2, PIK3CA, p-AKT, and AKT in individual tumors of the stable circ_6014 knockdown or negative control mice. (**I**) Quantitative statistics of protein expression levels in the two groups of mice. ^*^p < 0.05, ^**^p < 0.01, ^***^p < 0.001.

The xenograft assay was carried out to observe the ability of circ_6014 to promote tumorigenesis with the support of MDA-MB-231 cells infected with lentivirus expressing Plko.1-sh_NC or Plko.1-sh_circ_6014. The efficiency of infection in MDA-MB-231 cells was ensured by qRT-PCR (Figure 6A), which indicated that the expression of circ_6014 was significantly downregulated by shRNA. The results showed that the loss of function of circ_6014 could suppress tumor growth *in vivo* (Figure 6B–6D). Moreover, there was no significant difference in body weight between the two groups of nude mice over time (Figure 6E). Furthermore, the histograms of the relative expression of miR-885-3p and NTRK2 (Figure 6F, 6G) were consistent with the trend observed in the *in vitro* experiments. The relative protein expression of NTRK2 was decreased with the knockdown of circ_6014, while those of PIK3CA, p-AKT at threonine 308, and AKT were also decreased in the circ_6014 knockdown group (Figure 6H, 6I). Our results indicated that knockdown of circ_6014 can suppress BC tumor growth *in vivo*.

## DISCUSSION

In contrast to traditional noncoding RNAs, circRNAs are a group of highly conserved, covalent closed-looped RNAs with a reversed 5′ to 3′ end head-to-tail connection [25, 26]. The increased level of circRNF20 in BC indicates a poor clinical prognosis of patients [27]. These results suggest that circRNAs can be both oncogenes and potential therapeutic targets. Nevertheless, another novel circRNA, circ_6014, had upregulated expression in BC tumor samples compared with paracarcinoma samples and downregulated expression after the patients underwent the operation, but its mechanism in breast cancer is unknown.

The best-known mechanisms of circRNAs are serving as miRNA sponges [28], binding with RNA binding proteins (RBPs) [29] transferring miRNAs into cells [30], encoding proteins [31], etc. Among them, competitive endogenous binding to miRNAs has been the most studied. Circ_0060428 was shown to promote osteosarcoma cell proliferation through the miR-375/RBPJ axis [32]. In addition, the migration of hepatocellular carcinoma cells was found to be impeded by the circ_0003645/miR-1299/PI3K/mTOR pathway [33]. In this research, circ_6014 was predicted to have binding sites with miR-885-3p via bioinformatics analysis (Figure 3A). Moreover, miR-885-3p expression was apparently upregulated with circ_6014 interference but downregulated when exogenous circ_6014 was introduced. Moreover, the relative intensity of luciferase confirmed this hypothesis, which suggests that circ_6014 has a similar function as a miRNA sponge. Overexpressing circ_6014 can protect the inhibitory function of miR-885-3p in the proliferation of BC cells. Mechanistically, circ_6014 promoted proliferation by competing with miR-885-3p for binding.

One most common function of miRNAs is base-pairing with specific mRNAs [34], followed by cleavage of the mRNA into two pieces or a decrease in the translation of the mRNA [35, 36], e eventually silencing target mRNAs or regulating these molecules post-transcription [34, 37]. Several studies have been carried out on the function of miR-885-3p as a tumor suppressor, such as Cao J et al. proved that miR-885-3p can directly target the mitotic serine/threonine kinase Aurora A and help to increase the sensitivity to docetaxel chemotherapy in lung adenocarcinoma [38]. Moreover, this molecule can inhibit the process of gastric malignancy by combining with CDK4 [39]. Likewise, miR-885-3p decreased the activity of cellular proliferation and the ability of colony formation in BC, and miR-885-3p could affect the expression of CDK2/CCNE1 and CDK4/6/CCND1, which was consistent with the reported studies. Our *in vivo* results showed that loss of function of circ_6014 could suppress tumor growth and resulted in no significant difference in body weight between the two groups of nude mice over time. Furthermore, the histograms of the relative expression of miR-885-3p and NTRK2 were consistent with the trend observed in the *in vitro* experiments.

Additionally, bioinformatics analysis revealed possible binding sites between miR-885-3p and the 3′UTR of NTRK2 mRNA (Figure 4L). The two vectors were cotransfected into both HEK293T cells and MDA-MB-231 cells with mimics of miR-885-3p or its negative control. The results indicated that miR-885-3p could target NTRK2 in HEK293T and MDA-MB-231 cell lines. In addition, overexpression of miR-885-3p suppressed the mRNA and protein levels of NTRK2 in MDA-MB-231 and MCF-7 cells. The qRT-PCR results for the relative expression of NTRK2 in the miR-885-3p-overexpressing group also indicated that the miR-885-3p target NTRK2 showed strongly downregulated expression.

It has been reported that NTRK2 has significant associations with the side effects of breast cancer patients after chemotherapy [22]. Moreover, according to a bioinformatics analysis of differentially expressed genes between luminal B2 breast cancer tumor and nontumor tissues, NTRK2 participates in tumor-promoting pathways, focal adhesion, and ECM-receptor interactions and plays a leading role in tumorigenesis [40]. The PIK3/AKT pathway is tightly related to tumorigenesis, cellular progression, and transference in breast cancer [41, 42]. The relative expression level of NTRK2 decreased as expected when miR-885-3p was overexpressed or circ_6014 was knocked down. Furthermore, the relative expression of PIK3CA and phosphate AKT (threonine 308) showed the same trend as NTRK2, which is in agreement with the connection between NTRK2 and AKT predicted in a reliable database. Therefore, our results demonstrated that circ_6014 can sponge miR-885-3p to regulate the NTRK2 and PIK3/AKT pathways in breast cancer.

In summary, we found that circ_6014 may inhibit breast cancer, serve as a ceRNA and participate in the proliferation of breast cancer cells through the circ_6014/miR-885-3p/NTRK2-PIK3CA/AKT axis (Supplementary Figure 3), which suggests that circ_6014 can be a potential biomarker and clinical therapeutic target.

## MATERIALS AND METHODS

### Clinical specimen achievement and RNA extraction

16 pairs of matched tissues of breast cancer and para-carcinoma breast gland tissues were obtained from patients who underwent surgeries in the Department of Thyroid and Breast Surgery, Shanghai Tenth People’s Hospital (Shanghai, China). The approved ethics number is SHDSYY-2020-4531(Shanghai Tenth People’s Hospital). Likewise, 12 pairs of matched venous blood samples were drawn to sodium citrate-treated blood-collection tubes from patients from the same group of patients before and one day after the surgery. Every patient involved provided written informed consent, the experiments obtained ethical approval from the Ethical Committee of Shanghai Tenth People’s Hospital at the same time.

### Cell culture and transfection

Breast cancer cell lines MDA-MB-231 and MCF-7 were purchased from the Chinese Academy of Science at Shanghai (Shanghai, China). Human embryonic kidney (HEK) 293 T cell line was a kind gift from the Department of Laboratory Medicine, Shanghai Tenth People’s Hospital. The cells were cultured in Dulbecco’s Modified Eagle's medium (DMEM, Gibco, New York, USA) with 10% fetal bovine serum (FBS, Lonsera Lonsa Science SRL, Shanghai, China) and 1% penicillin-streptomycin (PS, Sigma-Aldrich; Merck KGaA, Darmstadt, Germany), in an incubator equipped with an artificial environment at 37°C with 5% CO_2_.

Small interfering RNAs (siRNAs), mimics and inhibitor of miR-885-3p and their negative control (NC), pcDNA3.1 (+) circRNA Mini Vector- circ_6014 and its empty vector for transient transfection were synthesized and purchased from Ibsbio (Shanghai, China). Their sequences were listed in Supplementary Table 4. Meanwhile, vectors were maintained in puncture bacteria and extracted with TIANprep Midi Plasmid Kit (TIANGEN, Beijing, China). All of the above were transfected with HieffTrans™ Liposomal Transfection Reagent (Yeasen, Shanghai, China) according to the manufacturer’s instructions with a concentration of 100 nM/mL for siRNA of circRNA, mimics, and inhibitor of miRNA, and a concentration of 2 μg/mL for the plasmid of pcDNA3.1(+) circRNA Mini Vector.

### Quantitative reverse transcription-polymerase chain reaction (qRT-PCR)

Total RNA was isolated from MDA-MB-231 and MCF-7 cells with TRIzol reagent (Invitrogen, Carlsbad, CA, USA) according to the manufacturer’s protocol, which cells were treated by siRNA, mimics, inhibitor or plasmid for 36 to 48 hours. cDNAs were generated by HiScript II Q Select RT SuperMix Kit (Vazyme; Nanjing, Jiangsu, China) followed by the manufacture’s protocol. qRT-PCR was performed on a 7900HT fast real-time PCR system (Applied Biosystems, Singapore). Primers used for detecting the abundance of circ_6014, miR-885-3p, NTRK2, and primers regarding internal calibrate the expression of hsa_circ_0006014, miRNA, and mRNA, which were respectively 18s rRNA, U6 snRNA, and β-ACTIN were listed in Supplementary Table 4.

### Actinomycin D treatment assay

Both breast cancer cell lines were respectively cultivated in 6-well plates till the cell confluence reached 90% to 100% per well. Then every well was changed with fresh full DMEM medium and exposed to 2 μg/mL Actinomycin D (KE1084, Kingmorn life science, Shanghai, China) for four, eight, or twelve hours. Then, total RNA was extracted from these cells and the expression of ILKAP mRNA and circ_6014 were detected by qRT-PCR, the details of RNA extraction and qRT-PCR are described at the third part of Materials and Methods.

### Methylthiazolyldiphenyl-tetrazolium bromide (MTT) proliferation assay

Treated breast cancer cells were transferred to a 96-well plate from a 6-well plate 24 hours after transfection. The cell proliferation rate was measured by testing the absorbance after dissolving the MTT (Beyotime, Shanghai, China) with dimethyl sulfoxide (DMSO, Sangon Biotech, Shanghai, China) according to the manufacturer’s instructions (Beyotime, Shanghai, China) for five days. A microplate spectrophotometer (BioTek, Winooski, VT, USA) was used to measure the absorbance of each sample at a wavelength of 490 nm.

### Colony formation assay

After transfected with siRNA, mimics, inhibitor, or plasmid for 24 hours, breast cancer cells were collected by centrifugation and prepared for single-cell suspensions. Then they were cultured in a new 6-well plate at a consistency of 600 cells per well for MDA-MB-231 cells and 800 cells per well for MCF-7 cells. Fresh complete medium was changed every three days. Approximately 10 days later, the bottom of the wells was washed with PBS twice, fixed with 4% paraformaldehyde for 10 minutes, stained with Crystal Violet and washed with ddH_2_O three times. Photos of the whole cloned cells of the per well were taken eventually.

### Transwell formation assay

After transfection mentioned above, breast cancer cells were collected by centrifugation and then they were cultured with 200 ul 2% FBS-DMEM in an upper-transwell cup with 30,000 cells per well for migration and 80,000 cells per well for invasion. The lower well was fulfilled with 500 ul 10% FBS-DMEM. Approximately 12 hours later, the bottom of the wells was washed with PBS twice, fixed with 4% paraformaldehyde for 10 minutes, stained with Crystal Violet and washed with ddH_2_O for three times and washed with ddH_2_O three times. Photos of the whole bottom migrated or invasive cells of the well were taken eventually.

### Cell cycle analysis

The MDA-MB-231 and MCF-7 cells transfected with siRNA, mimics, or plasmid for 36 hours subsequently. After that, the cells were respectively collected and washed with ice-bathed PBS twice, fixed in ice-bathed 75% ethanol, and stored at 4°C for 24 hours. Finally, after washing with ice-bathed PBS twice, both cells were stained with propidium iodide (PI)/ RNase A mixture (Beyotime, Shanghai, China) at 37°C for 30 minutes and analyzed by flow cytometry (BD Pharmingen Franklin Lakes, NJ, USA).

### Western blot assay

Total proteins of breast cancer cells MDA-MB-231 and MCF-7 were extracted 72 hours after transfection. They were washed with precooled PBS three times and lysed for 30 minutes on ice with RIPA lysis buffer (Beyotime, Shanghai, China), which was pre-mixed with InStab™ phosphatase inhibitor cocktail (Yeasen, Shanghai, China) according to the manufacturer’s protocol. Then the protein concentration was detected by BCA Protein Assay Kit (Beyotime, Shanghai, China) and dyed in 6× sodium dodecyl sulfate (SDS) loading buffer (Beyotime, Shanghai, China), denatured at 100°C for 10 minutes finally. Subsequently, 60 microliters of denatured protein samples were separated on a 7% SDS-PAGE gel and then electrophoretically transferred onto 0.45-μm nitrocellulose membranes (Beyotime, Shanghai, China). The membranes were blocked with 5% BSA for (Sigma-Aldrich; Merck KGaA, Darmstadt, Germany) an hour and incubated with specific primary antibodies at 4°C overnight. Afterwards, the membranes were washed with PBS joined with Tween 20 (1000:1) to gotten rid of the rest of primary antibodies, then incubated with fluorescently conjugated secondary anti-rabbit or anti-mouse antibodies. The target protein bands were scanned by an Odyssey Scanning System (LI-COR Biosciences, Lincoln, NE, USA) at last. Details for primary and secondary antibodies used were listed in Supplementary Table 5.

### Dual-luciferase reporter assay

Specific fragments of circ_6014 and NTRK2 3′-UTR containing the predicted binding site of miR-885-3p were inserted into the dual luciferase plasmid psiCHECK2 vector (Ibsbio, Shanghai, China) at the downstream of the *Renilla* luciferase gene to construct pmirGLO- circ_6014 vector and pmirglo-NTRK2 3′-UTR vector.

HEK 293T cells and MDA-MB-231 cells were transiently co-transfected with 0.5 micrograms of psiCHECK2 vectors constructed above in addition to 50 nmol of miR-885-3p mimics or its negative control using Hieff Trans™ Liposomal Transfection Reagent (Yeasen, Shanghai, China). 48–72 hours later, total protein from cell lysates was collected and centrifuged. Afterward, firefly and renilla signals were determined using a Dual-Lumi™ Luciferase Reporter Gene Assay Kit (Beyotime, Shanghai, China). Firefly signals were normalized to the corresponding renilla signals while the results were offered as the ratio of firefly to renilla signals.

### Stable cell line construction

The shRNA of hsa_circ_0006014 (Plko.1-puro-GFP- circ_6014) and its empty vector for stable transfection were synthesized by Ibsbio (Shanghai, China). They were separately constructed into lentivirus purchased from Zorin (Shanghai, China) according to the manufacturer's protocol.

MDA-MB-231 cells (2.0 × 10^5^/well) were cultured in 6-well plates till they reached 90% to 100% confluence for lentivirus infection. The eventual volume of the mixture of the solution was two milliliters per hsa_circ_0006014 knock-down well and hsa_circ_0006014 knock-down negative-control well, blank wells without any disposing were also set. Hsa_circ_0006014 knocked-down MDA-MB-231 cells and its negative-control cells were amplified when the cells in the blank wells were demised and get the hsa_circ_0006014 stable knockdown cell lines.

### Tumor growth in nude mice

Weighing more than 20 grams and four-week-old athymic female nude mice (*n* = 10) were obtained from LinChang (Shanghai, China) and maintained in the SPF animal house of the North Campus of Tongji University. MDA-MB-231 Plko.1-sh_NC or Plko.1-sh_circ_6014 cells (1.5 × 10^6^) were subcutaneous injections under the left side of the second pair of mammary glands in nude mice. The tumor volume was measured every three days and calculated by the following formula: Volume (mm^3^) = Length (mm) × Width^2^ (mm^2^) × 0.5. After 30 days, the mice were sacrificed, and the tumors were collected for weighing. All the operations were conducted under the permission of the ethical committee of Shanghai Tenth People’s Hospital.

### Statistical analysis

SPSS v20.0 software (SPSS, Inc., Chicago, IL, USA) was used for Pearson’s χ^2^ test to analyze the associated expression levels between circ_6014 and clinical-pathological parameter features of patients. ModFitLT 3.2 software (Verity Software House, Inc., Topsham, ME, USA) was used for analyzing the percentage of cell cycle distribution of G0/G1, S, and G2/M phases. GraphPad Prism v6.0 software (GraphPad, San Diego, CA, USA) was used for all statistical analyses. All quantitative results included in this study were representations of at least three duplicates in three separated experiments, then presented as mean ± standard (mean ± SD), in case of specially intents like the tumor volumes and weights of the xenograft assay were represented as the mean ± SEM of five mice. Moreover, student’s *t*-test and one-way ANOVA were used to value the data, and differences regarded significantly when *p* < 0.05.

## Supplementary Materials

Supplementary Figures

Supplementary Tables

## References

[1] World Health Organization. Latest Global Cancer Data: Cancer Burden Rises to 19.3 Million New Cases and 10.0 Million Cancer Deaths in 2020. 2020. https://www.iarc.who.int/news-events/latest-global-cancer-data-cancer-burden-rises-to-19-3-million-new-cases-and-10-0-million-cancer-deaths-in-2020/

[2] Sung H, Ferlay J, Siegel RL, Laversanne M, Soerjomataram I, Jemal A, Bray F. Global Cancer Statistics 2020: GLOBOCAN Estimates of Incidence and Mortality Worldwide for 36 Cancers in 185 Countries. CA Cancer J Clin. 2021; 71:209–49. 10.3322/caac.21660 33538338

[3] Modi ND, Tan JQE, Rowland A, Koczwara B, Abuhelwa AY, Kichenadasse G, McKinnon RA, Wiese MD, Sorich MJ, Hopkins AM. The obesity paradox in early and advanced HER2 positive breast cancer: pooled analysis of clinical trial data. NPJ Breast Cancer. 2021; 7:30. 10.1038/s41523-021-00241-9 33753745 PMC7985140

[4] Nindrea RD, Aryandono T, Lazuardi L, Dwiprahasto I. Family History of Breast Cancer and Breast Cancer Risk between Malays Ethnicity in Malaysia and Indonesia: A Meta-Analysis. Iran J Public Health. 2019; 48:198–205. 10.18502/ijph.v48i2.814 31205873 PMC6556193

[5] Prusty RK, Begum S, Patil A, Naik DD, Pimple S, Mishra G. Knowledge of symptoms and risk factors of breast cancer among women: a community based study in a low socio-economic area of Mumbai, India. BMC Womens Health. 2020; 20:106. 10.1186/s12905-020-00967-x 32423488 PMC7236367

[6] Kvaskoff M, Mahamat-Saleh Y, Farland LV, Shigesi N, Terry KL, Harris HR, Roman H, Becker CM, As-Sanie S, Zondervan KT, Horne AW, Missmer SA. Endometriosis and cancer: a systematic review and meta-analysis. Hum Reprod Update. 2021; 27:393–420. 10.1093/humupd/dmaa045 33202017

[7] Huo Y, Selenica P, Mahdi AH, Pareja F, Kyker-Snowman K, Chen Y, Kumar R, Da Cruz Paula A, Basili T, Brown DN, Pei X, Riaz N, Tan Y, et al. Genetic interactions among Brca1, Brca2, Palb2, and Trp53 in mammary tumor development. NPJ Breast Cancer. 2021; 7:45. 10.1038/s41523-021-00253-5 33893322 PMC8065161

[8] Haddad JM, Robison K, Beffa L, Laprise J, ScaliaWilbur J, Raker CA, Clark MA, Hofstatter E, Dalela D, Brown A, Bradford L, Toland M, Stuckey A. Family planning in carriers of BRCA1 and BRCA2 pathogenic variants. J Genet Couns. 2021; 30:1570–81. 10.1002/jgc4.1423 33904624

[9] Hsu MT, Coca-Prados M. Electron microscopic evidence for the circular form of RNA in the cytoplasm of eukaryotic cells. Nature. 1979; 280:339–40. 10.1038/280339a0 460409

[10] Chen L, Shan G. CircRNA in cancer: Fundamental mechanism and clinical potential. Cancer Lett. 2021; 505:49–57. 10.1016/j.canlet.2021.02.004 33609610

[11] Pan G, Mao A, Liu J, Lu J, Ding J, Liu W. Circular RNA hsa_circ_0061825 (circ-TFF1) contributes to breast cancer progression through targeting miR-326/TFF1 signalling. Cell Prolif. 2020; 53:e12720. 10.1111/cpr.12720 31961997 PMC7048212

[12] Liu T, Ye P, Ye Y, Lu S, Han B. Circular RNA hsa_circRNA_002178 silencing retards breast cancer progression via microRNA-328-3p-mediated inhibition of COL1A1. J Cell Mol Med. 2020; 24:2189–201. 10.1111/jcmm.14875 31957232 PMC7011152

[13] Inoue J, Inazawa J. Cancer-associated miRNAs and their therapeutic potential. J Hum Genet. 2021; 66:937–45. 10.1038/s10038-021-00938-6 34088973

[14] Shen S, Song Y, Zhao B, Xu Y, Ren X, Zhou Y, Sun Q. Cancer-derived exosomal miR-7641 promotes breast cancer progression and metastasis. Cell Commun Signal. 2021; 19:20. 10.1186/s12964-020-00700-z 33618729 PMC7898766

[15] Mansoori B, Duijf PHG, Mohammadi A, Safarzadeh E, Ditzel HJ, Gjerstorff MF, Cho WC, Baradaran B. MiR-142-3p targets HMGA2 and suppresses breast cancer malignancy. Life Sci. 2021; 276:119431. 10.1016/j.lfs.2021.119431 33785332

[16] Shi Y, Liu Z, Lin Q, Luo Q, Cen Y, Li J, Fang X, Gong C. MiRNAs and Cancer: Key Link in Diagnosis and Therapy. Genes (Basel). 2021; 12:1289. 10.3390/genes12081289 34440464 PMC8395027

[17] Zhao Y, Jin LJ, Zhang XY. Exosomal miRNA-205 promotes breast cancer chemoresistance and tumorigenesis through E2F1. Aging (Albany NY). 2021; 13:18498–514. 10.18632/aging.203298 34292880 PMC8351670

[18] Li M, Zou X, Xia T, Wang T, Liu P, Zhou X, Wang S, Zhu W. A five-miRNA panel in plasma was identified for breast cancer diagnosis. Cancer Med. 2019; 8:7006–17. 10.1002/cam4.2572 31568692 PMC6853814

[19] Adams I, Yang T, Longo FM, Katz DM. Restoration of motor learning in a mouse model of Rett syndrome following long-term treatment with a novel small-molecule activator of TrkB. Dis Model Mech. 2020; 13:dmm044685. 10.1242/dmm.044685 33361117 PMC7710018

[20] Weiss LM, Funari VA. NTRK fusions and Trk proteins: what are they and how to test for them. Hum Pathol. 2021; 112:59–69. 10.1016/j.humpath.2021.03.007 33794242

[21] Solomon JP, Linkov I, Rosado A, Mullaney K, Rosen EY, Frosina D, Jungbluth AA, Zehir A, Benayed R, Drilon A, Hyman DM, Ladanyi M, Sireci AN, Hechtman JF. NTRK fusion detection across multiple assays and 33,997 cases: diagnostic implications and pitfalls. Mod Pathol. 2020; 33:38–46. 10.1038/s41379-019-0324-7 31375766 PMC7437403

[22] Young EE, Kelly DL, Shim I, Baumbauer KM, Starkweather A, Lyon DE. Variations in COMT and NTRK2 Influence Symptom Burden in Women Undergoing Breast Cancer Treatment. Biol Res Nurs. 2017; 19:318–28. 10.1177/1099800417692877 28205449 PMC5873314

[23] Fresno Vara JA, Casado E, de Castro J, Cejas P, Belda-Iniesta C, González-Barón M. PI3K/Akt signalling pathway and cancer. Cancer Treat Rev. 2004; 30:193–204. 10.1016/j.ctrv.2003.07.007 15023437

[24] Akbarzadeh M, Mihanfar A, Akbarzadeh S, Yousefi B, Majidinia M. Crosstalk between miRNA and PI3K/AKT/mTOR signaling pathway in cancer. Life Sci. 2021; 285:119984. 10.1016/j.lfs.2021.119984 34592229

[25] Zhang Y, Zhang Y, Li X, Zhang M, Lv K. Microarray analysis of circular RNA expression patterns in polarized macrophages. Int J Mol Med. 2017; 39:373–9. 10.3892/ijmm.2017.2852 28075448 PMC5358696

[26] Memczak S, Jens M, Elefsinioti A, Torti F, Krueger J, Rybak A, Maier L, Mackowiak SD, Gregersen LH, Munschauer M, Loewer A, Ziebold U, Landthaler M, et al. Circular RNAs are a large class of animal RNAs with regulatory potency. Nature. 2013; 495:333–8. 10.1038/nature11928 23446348

[27] Cao L, Wang M, Dong Y, Xu B, Chen J, Ding Y, Qiu S, Li L, Karamfilova Zaharieva E, Zhou X, Xu Y. Circular RNA circRNF20 promotes breast cancer tumorigenesis and Warburg effect through miR-487a/HIF-1α/HK2. Cell Death Dis. 2020; 11:145. 10.1038/s41419-020-2336-0 32094325 PMC7039970

[28] Jia Q, Ye L, Xu S, Xiao H, Xu S, Shi Z, Li J, Chen Z. Circular RNA 0007255 regulates the progression of breast cancer through miR-335-5p/SIX2 axis. Thorac Cancer. 2020; 11:619–30. 10.1111/1759-7714.13306 31962380 PMC7049509

[29] Wilusz JE, Sharp PA. Molecular biology. A circuitous route to noncoding RNA. Science. 2013; 340:440–1. 10.1126/science.1238522 23620042 PMC4063205

[30] Hentze MW, Preiss T. Circular RNAs: splicing's enigma variations. EMBO J. 2013; 32:923–5. 10.1038/emboj.2013.53 23463100 PMC3616293

[31] Wu X, Xiao S, Zhang M, Yang L, Zhong J, Li B, Li F, Xia X, Li X, Zhou H, Liu D, Huang N, Yang X, et al. A novel protein encoded by circular SMO RNA is essential for Hedgehog signaling activation and glioblastoma tumorigenicity. Genome Biol. 2021; 22:33. 10.1186/s13059-020-02250-6 33446260 PMC7807754

[32] Cao J, Liu XS. Circular RNA 0060428 sponges miR-375 to promote osteosarcoma cell proliferation by upregulating the expression of RPBJ. Gene. 2020; 740:144520. 10.1016/j.gene.2020.144520 32130980

[33] Yu Q, Dai J, Shu M. Retraction: Hsa_circ_0003645 shows an oncogenic role by sponging microRNA-1299 in hepatocellular carcinoma cells. J Clin Lab Anal. 2020; 34:e23249. 10.1002/jcla.23249 32108372 PMC7307333

[34] Bartel DP. MicroRNAs: target recognition and regulatory functions. Cell. 2009; 136:215–33. 10.1016/j.cell.2009.01.002 19167326 PMC3794896

[35] Bertolazzi G, Benos PV, Tumminello M, Coronnello C. An improvement of ComiR algorithm for microRNA target prediction by exploiting coding region sequences of mRNAs. BMC Bioinformatics. 2020 (Suppl 8); 21:201. 10.1186/s12859-020-3519-5 32938407 PMC7493982

[36] Fabian MR, Sonenberg N, Filipowicz W. Regulation of mRNA translation and stability by microRNAs. Annu Rev Biochem. 2010; 79:351–79. 10.1146/annurev-biochem-060308-103103 20533884

[37] Galván-Román JM, Lancho-Sánchez Á, Luquero-Bueno S, Vega-Piris L, Curbelo J, Manzaneque-Pradales M, Gómez M, de la Fuente H, Ortega-Gómez M, Aspa J. Usefulness of circulating microRNAs miR-146a and miR-16-5p as prognostic biomarkers in community-acquired pneumonia. PLoS One. 2020; 15:e0240926. 10.1371/journal.pone.0240926 33095833 PMC7584179

[38] Cao J, Geng J, Chu X, Wang R, Huang G, Chen L. miRNA-885-3p inhibits docetaxel chemoresistance in lung adenocarcinoma by downregulating Aurora A. Oncol Rep. 2019; 41:1218–30. 10.3892/or.2018.6858 30431113

[39] Lin Z, Zhou Z, Guo H, He Y, Pang X, Zhang X, Liu Y, Ao X, Li P, Wang J. Long noncoding RNA gastric cancer-related lncRNA1 mediates gastric malignancy through miRNA-885-3p and cyclin-dependent kinase 4. Cell Death Dis. 2018; 9:607. 10.1038/s41419-018-0643-5 29789536 PMC5964145

[40] Wang J, Du Q, Li C. Bioinformatics analysis of gene expression profiles to identify causal genes in luminal B2 breast cancer. Oncol Lett. 2017; 14:7880–8. 10.3892/ol.2017.7256 29250180 PMC5727610

[41] Peng B, Li C, He L, Tian M, Li X. miR-660-5p promotes breast cancer progression through down-regulating TET2 and activating PI3K/AKT/mTOR signaling. Braz J Med Biol Res. 2020; 53:e9740. 10.1590/1414-431X20209740 33146288 PMC7643928

[42] Jouali F, Marchoudi N, Talbi S, Bilal B, El Khasmi M, Rhaissi H, Fekkak J. Detection of PIK3/AKT pathway in Moroccan population with triple negative breast cancer. BMC Cancer. 2018; 18:900. 10.1186/s12885-018-4811-x 30227836 PMC6145190

