# Supplementary material for: Circular RNA_0006014 promotes breast cancer progression through sponging miR-885-3p to regulate NTRK2 and PIK3/AKT pathway
Source: Aging (Albany NY). 2022 Apr 5;14(7):3105–28. doi: 10.18632/aging.203996 (PMC9037253; doi:10.18632/aging.203996)
Supplement: Supplementary Figures [file aging-14-203996-s001.pdf]

SUPPLEMENTARY FIGURES

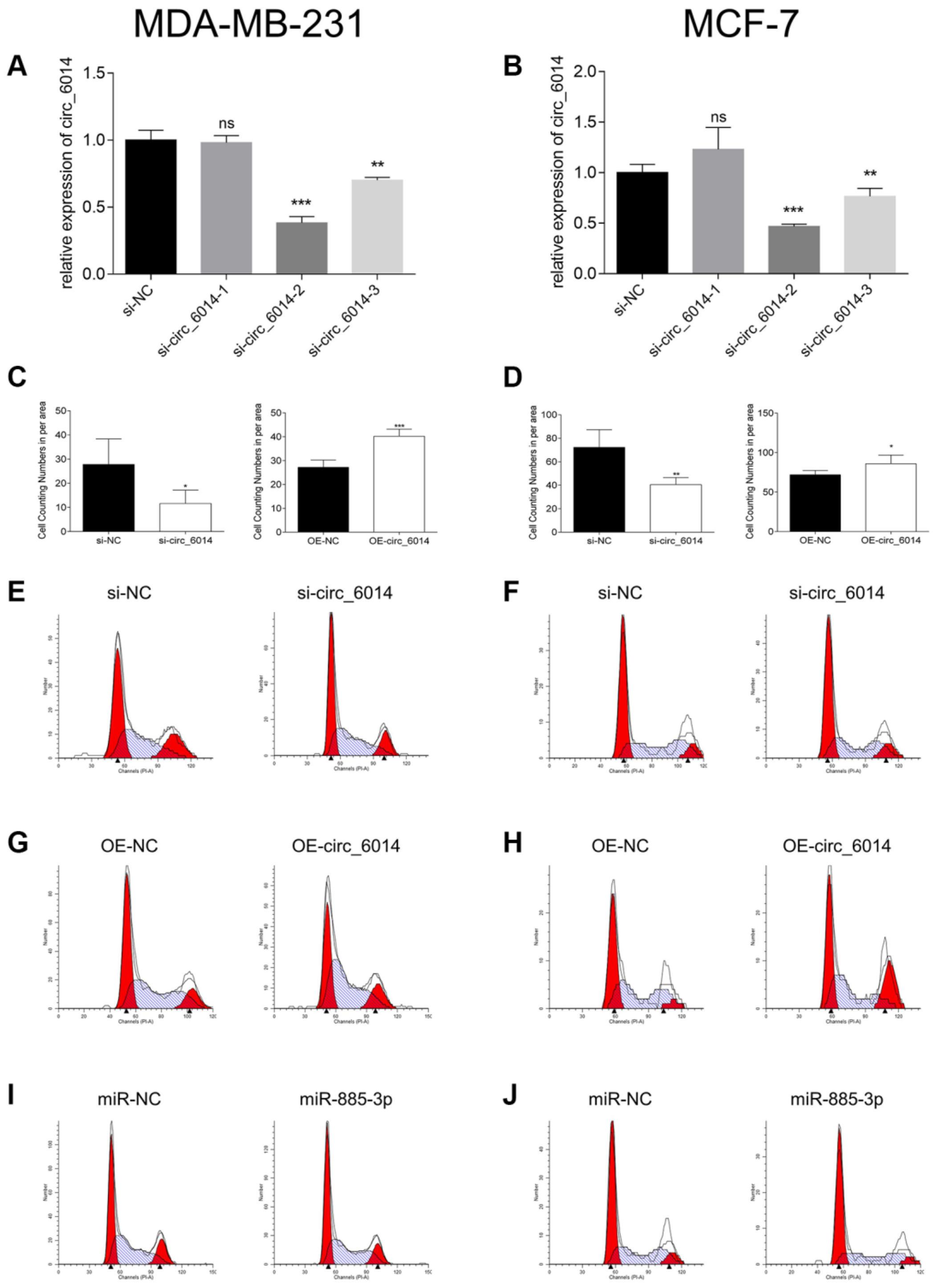

Supplementary Figure 1. Complementary biological functions of circ_6014 in breast cancer cells. (**A**, **B**) Expression levels of circ_6014 in both MDA-MB-231 and MCF-7 cells after transfection with three small interfering RNAs synthesized by Ibsbio (Shanghai, China). (**C**, **D**) Cell counting numbers of per area of the colony formation assay with decreased or overexpressed levels of circ_6014. (**E–J**) Original flow cytometry graphs of the cell cycle assay with decreased or overexpressed levels of circ_6014, or increased levels of miR-885-3p. $^{*}p < 0.05$, $^{**}p < 0.01$, $^{***}p < 0.001$.

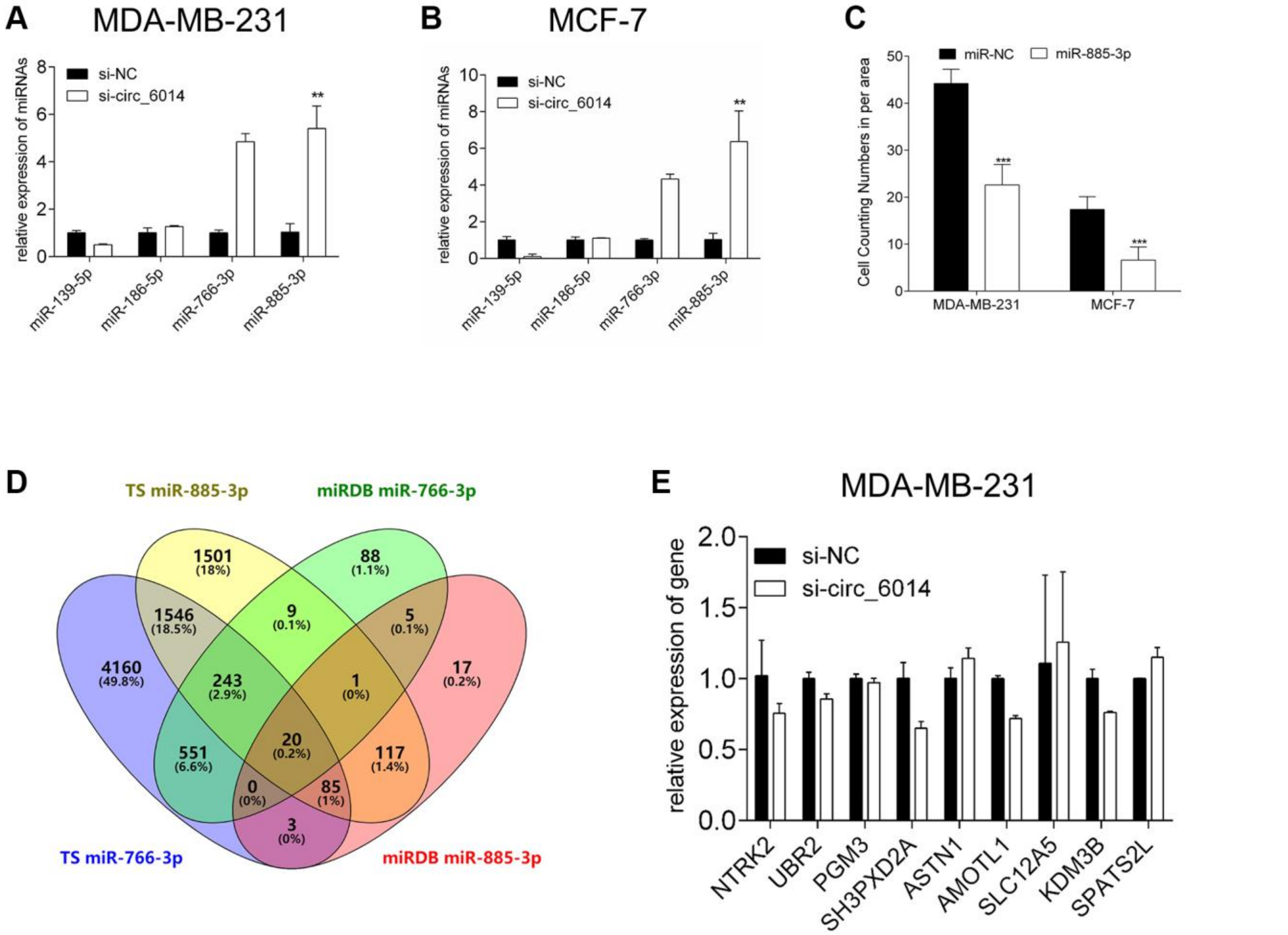

F

	Predicted consequential pairing of target region (top) and miRNA (bottom)	Site type	Context++ score	Context++ score percentile	Weighted context++ score	Conserved branch length	P_{CT}
Position 52-58 of NTRK2 3' UTR<br>hsa-miR-885-3p	5′ ...UGCUUGUUGGUUGAUGCUGCCAU...<br>|||||| 3′ AUAGGUGAUGUGGGGCGACGGA	7mer-A1	-0.11	76	-0.11	0	N/A
Position 2837-2843 of NTRK2 3' UTR<br>hsa-miR-885-3p	5′ ...AACAAAAGUCUGUGGGCUGCCAG...<br>|||||| 3′ AUAGGUGAUGUGGGGCGACGGA	7mer-A1	-0.06	60	-0.06	0.043	N/A
Position 3219-3225 of NTRK2 3' UTR<br>hsa-miR-885-3p	5′ ...UUGGCCCAGUCAGGUGCUGCCAG...<br>|||||| 3′ AUAGGUGAUGUGGGGCGACGGA	7mer-A1	-0.04	47	-0.04	0.016	N/A
Position 4441-4447 of NTRK2 3' UTR<br>hsa-miR-885-3p	5′ ...CCAUGAAUUGAAUUUGCUGCCAG...<br>|||||| 3′ AUAGGUGAUGUGGGGCGACGGA	7mer-A1	-0.12	78	-0.12	0.043	N/A
Position 4451-4457 of NTRK2 3' UTR<br>hsa-miR-885-3p	5′ ...AAUUUGCUGCCAGGUGCUGCCAG...<br>|||||| 3′ AUAGGUGAUGUGGGGCGACGGA	7mer-A1	-0.09	71	-0.09	0.016	N/A

Supplementary Figure 2. Additional information of miRNAs and mRNAs. (**A**, **B**) Relative expressions of four miRNAs filtrated from four databases in both MDA-MB-231 and MCF-7 cells. (**C**) Cell counting numbers of per area of the colony formation assay with increased levels of miR-885-3p. (**D**) The Venn diagram of potential mRNAs that have binding sites with miR-885-3p and miR-766-3p according to TargetScan and miRDB. (**E**) Relative expressions of the nine candidates in circ_6014 downregulated MDA-MB-231 cells. (**F**) Five probable binding sites between miR-885-3p and the 3'-UTR region of NTRK2 mRNA. $^{*}p < 0.05$, $^{**}p < 0.01$, $^{***}p < 0.001$.

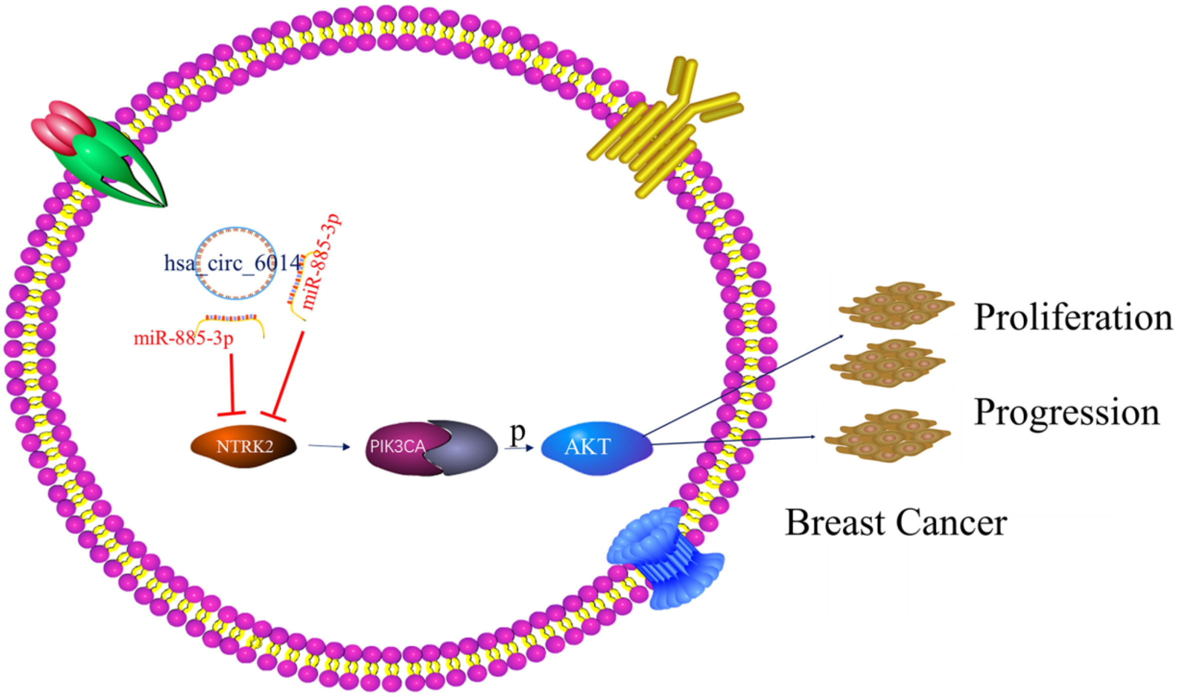

Supplementary Figure 3. Simulated mechanism diagram of the circ_6014/miR-885-3p/NTRK2-PIK3CA/AKT axis in breast cancer.
