# Supplementary material for: Circular RNA_0006014 promotes breast cancer progression through sponging miR-885-3p to regulate NTRK2 and PIK3/AKT pathway
Source: Aging (Albany NY). 2022 Apr 5;14(7):3105–28. doi: 10.18632/aging.203996 (PMC9037253; doi:10.18632/aging.203996)
Supplement: Supplementary Tables [file aging-14-203996-s002.pdf]

SUPPLEMENTARY TABLES

Supplementary Table 1. MiRNAs that have potential binding sites with hsa_circ_0006014 predicted by circular RNA interactome, ENCORI, miRanda, and RNA hybrid databases.

	Circular RNA Interactome	StarBase	MiRanda	RNA hybrid
miR-1236	√			
miR-1238	√			
miR-1247-5p	√		√	√
miR-1251-5p	√		√	√
miR-1304-5p	√		√	
miR-139-5p	√	√		√
miR-142-5p	√			
miR-149-5p	√			√
miR-182-5p	√	√	√	
miR-186-5p	√	√	√	
miR-409-3p	√		√	
miR-485-3p	√			
miR-488	√			
miR-545-3p	√		√	
miR-548p	√		√	
miR-571	√		√	
miR-572	√		√	
miR-616-3p	√		√	√
miR-651	√			
miR-671-5p	√			
miR-766-3p	√		√	√
miR-885-3p	√		√	√
miR-934	√			√
miR-940	√		√	

Supplementary Table 2. The biological functions of the 8 miRNAs which coincidently predicted in three out of four databases and half of them have clear evidence that they can suppress cancer.

	The total article associated with cancer (update to 202110114)	Tumor Promoter	Evidence	Tumor Suppressor	Evidence
miR-1247-5p	8	+	PMID:25731699;	+	PMID:30249392;<br>PMID:30175411;<br>PMID:28586038;<br>PMID:30967263;
miR-1251-5p	2	+	PMID:31278033;<br>PMID:31918285	−	
miR-139-5p	192	+	PMID:29516499;	+	PMID:29335523;<br>PMID:29618950;<br>PMID:31571923;<br>PMID:29673587;<br>PMID:30710422;etc.
miR-182-5p	128	+	PMID:29361949;<br>PMID:30205384;<br>PMID:30285878;<br>PMID:30666836;etc.	+	PMID:30037856;<br>PMID:30285878;
miR-186-5p	56	+	PMID:30981721;	+	PMID:30443183;<br>PMID:30897321;<br>PMID:29325758;<br>PMID:30662805;etc.
miR-616-3p	3	+	PMID:29777710	−	
miR-766-3p	12	−		+	PMID:30145863;<br>PMID:28657135;<br>PMID:31330234;
miR-885-3p	11	−		+	PMID:30784279;<br>PMID:24882581;etc.

Supplementary Table 3. Nine mRNAs marked red were candidates from the four middle coincident areas which were proved to promote the progression of cancer from published articles.

20 common elements in "targetscan766", "targetscan885", "mirdb766" and "mirdb885":	9 common elements in "targetscan885" and "mirdb766":	1 common element in "targetscan885", "mirdb766" and "mirdb885":
VAPB	FBLN1	NTRK2
SLC8B1	FAM120C	
SH3PXD2A	DSG1	
ASTN1	RNASEL	
ZNF783	UBR2	
ERC1	PGM3	
AMOTL1	TRIM35	
PAN3	ASPHD2	
TSHZ2	PIAS2	
SLC12A5		
FAM126B		
BTBD9		
KDM3B		
SCMH1		
CDK6		
ZHX3		
SMIM12		
GGCX		
SPATS2L		
CELF3		

Supplementary Table 4. Sequences for transient transfection RNAs and primers for circular RNA, micro RNAs, and mRNAs.

Name		Sequence (5′–3′)
siRNA-hsa_circ_0006014-1	Sense	CAAUGACAGUACUCGGGUUTT
	Antisense	AACCCGAGUACUGUCAUUGTT
siRNA-hsa_circ_0006014-2	Sense	ACAGUACUCGGGUUUCAUATT
	Antisense	UAUGAAACCCGAGUACUGUTT
siRNA-hsa_circ_0006014-3	Sense	CCAAUGACAGUACUCGGGUTT
	Antisense	ACCCGAGUACUGUCAUUGGTT
siRNA-NC	Sense	UUCUCCGAACGUGUCACGUTT
	Antisense	ACGUGAACGUUCGGAGAATT
mimics of miR-885-3p	Sense	AGGCAGCGGGGUGUAAGUGGAUA
	Antisense	UCCACUACACCCCGCUGCCUUU
mimics of NC	Sense	UCACAACCUCCUAGAAAGAGUAGA
	Antisense	UACUCUUUCUAGGAGGUUGUGAUU
inhibitor of miR-885-3p		UAUCCACUACACCCCGCUGCCU
inhibitor of NC		UCUACUCUUUCUAGGAGGUUGUGA
18S	Forward	CAGCCACCCGAGATTGAGCA
	Reverse	TAGTAGCGACGGGCGGTGTG
hsa_circ_0006014	Forward	AAGAGCGGATGAGGATACAG
	Reverse	AACAGCAAAATATGAAACCC
	RT	AAAAATATGGAACGCTCACGAATTTG
U6	Forward	GTGCTCGCTTCGGCAGCACATATAC
	Reverse	AAAAATATGGAACGCTCACGAATTTG
miR-139-5p	Forward	CTCTGCTCTACAGTGCACGTGTC
	Reverse	TATGGTTGTTCTCGACTCCTTCAC
miR-186-5p	Forward	CTCCAACGCAAAGAATTCTCC
	Reverse	TATGCTTGTTCTCGTCTCTGTGTC
miR-766-3p	Forward	AATACACTCCAGCCCCACAGC
	Reverse	ATTCCATGTTGTCCACAGTCTCC
miR-885-3p	Forward	CAGTAGGCAGCGGGGTGTAG
	Reverse	TATGCTTGTTCTCGTCTCTGTGTC
β-ACTIN	Forward	CCTCGCCTTTGCCGATCC
	Reverse	GGATCTTCATGAGGTAGTCAGTC
NTRK2	Forward	GCAATCCATTTACATGCTCCTGT
	Reverse	CATATTAGGAACCGGATCACCTG
UBR2	Forward	GTACCAGCATTTAGCCCACTATG
	Reverse	TGCAAGAATATGTAGGCTCTCCT
PGM3	Forward	GCAGAGAGTGCTTATTGACATCA
	Reverse	TGTGAAAGTTTCTCACTGCTGG
SH3PXD2A	Forward	AGACTATCTACCGGAGGTACAGC
	Reverse	GCCACCTTCAATGGGAAACTT
ASTN1	Forward	ACGACCTGAGCATCATGCAC
	Reverse	CACCACGACCATTTCTCCCG
AMOTL1	Forward	GAAACATCTGCTTTGACGGTGG
	Reverse	GAAGTTTGGGGAGTGGAAGTTAC
SLC12A5	Forward	GCCACCATGCTAAACAACCTG
	Reverse	GGTTCTCAGGAGTAGATGGTGAT

KDM3B	Forward	GCTTCGGTTCCTGTCAGATG
	Reverse	GACCTCGGCTGAATATCTCTTG
SPATS2L	Forward	CAACGCTGCACCGTTTCTCTA
	Reverse	GACGAGCAGTCAGGATTTCCA

Supplementary Table 5. Details for primary and secondary antibodies.

Name	Dilution Ratio	Company
TrkB	1:500	Abcam, Cambridge, UK
PIK3CA	1:2000	Abcam, Cambridge, UK
pan-AKT	1:500	Abcam, Cambridge, UK
pan-AKT (T308)	1:500	Abcam, Cambridge, UK
CDK2	1:500	Bioworld, Minnesota, USA
Cyclin E1	1:1000	CST, Massachusetts, USA
CDK4	1:1000	CST, Massachusetts, USA
CDK6	1:50000	Abcam, Cambridge, UK
Cyclin D1	1:10000	Abcam, Cambridge, UK
PCNA	1:1000	Abclonal, WuHan, China
β-actin	1:1000	Santa Cruz Biotechnology, CA, USA
Anti-rabbit secondary fluorescent antibody	1:1000	Santa Cruz Biotechnology, CA, USA
Anti-mouse secondary fluorescent antibody	1:1000	Santa Cruz Biotechnology, CA, USA
